# A scoping review of the existing evidence linking school food procurement contract type with school food provision

**DOI:** 10.1371/journal.pone.0305685

**Published:** 2025-03-19

**Authors:** Nicola Nixon, Maria Bryant, Laura Sheard, Louise Padgett, Bob Doherty

**Affiliations:** 1 Department of Health Sciences, University of York, York, United Kingdom; 2 Hull York Medical School, University of York, York, United Kingdom; 3 School for Business and Society, University of York, York, United Kingdom; Federal University of Minas Gerais: Universidade Federal de Minas Gerais, BRAZIL

## Abstract

**Objective:**

School food and catering constitutes the largest area of public sector food spend in the UK, with the potential to influence health on a population scale. This review sought to understand and map the existing evidence linking school meals contracts for food procurement with the quality of food provided and health and academic outcomes for school children.

**Design:**

A scoping review of the peer reviewed and grey literature published between 1988 and 2023 was conducted. The strategy searched in Medline, Web of Science, Scopus, ERIC and Google, using key words related to population, exposure and outcomes.

**Setting:**

UK and International.

**Participants:**

School meal services.

**Results:**

Thirty documents were included representing 16 papers, 3 books and 11 reports. Documents revealed a complex and fragmented school meal provision system and inconsistent evidence relating to the outcomes of interest. Most studies focused on sustainability or nutrition/ guideline compliance and the main types of food providers discussed were commercial contractors, local authorities and in house catering. However, there was a lack of clarity in contract specifications and definitions of quality and concerns over compliance monitoring and financial viability impacting quality. We found no substantial body of peer reviewed research linking school food procurement contract type with food quality or outcomes of interest.

**Conclusions:**

The lack of research in this area (and conflicting findings) meant that it was impossible to draw robust conclusions on the benefits of using any particular contract provision type over another. Given the magnitude of public sector spending and the need for urgent improvements to the dietary health of the nation, this presents a significant gap in our knowledge.

## Introduction

The social and economic cost of poor dietary health in the UK is overwhelming, with markers of diet related chronic disease, such as overweight and obesity, evident as early as the primary school years [1–3]. Poor nutrition and unhealthy diets are associated with adverse outcomes for children, impacting their future health, economic prospects, and contribution to society [3,4]. Approximately 8% of annual UK healthcare spend, £18 billion, is spent on diet related illness [3]. Research has shown a correlation between fruit and vegetable consumption and educational attainment, with improved dietary quality and satiety for children associated with better school attendance and academic results [5,6]. Addressing the imbalance in dietary health is a key theme of the government’s levelling up white paper [7].

Research also demonstrates that dietary habits formed in childhood impact health outcomes in adulthood and that the risk of obesity can transfer between generations [4,8,9]. This would justify support for better dietary health and wellbeing during childhood to address current population health issues [4,10]. In the UK, 9 million children attend school, where they consume 30% to 50% of their daily food intake [2,11,12]. Therefore, school meals are an ideal method of providing nutritious, tasty food at this key stage in children’s mental, educational, and physical development, and an opportunity to impact population health [11,13–15]. The importance of quality and accessibility of school meals, particularly for children in the most deprived areas has been highlighted in research and by advocates [3,5,16]. However, there is no standard definition for dietary quality.

At approximately £700m, school food catering is the largest area of public sector food spend in the UK, accounting for 29% of the annual £2.4bn expenditure [17–19]. The Food and Agriculture Organisation refers to public food procurement initiatives, including school meals, as ‘game-changers’ due to their ability to influence food consumption, healthy diets, and more sustainable food systems [20].

School meal provision in the UK changed considerably in the 1980’s with the devolution of responsibility from local authorities (i.e., local government) to individual schools and the introduction of compulsory competitive tendering, opening school meal provision to the private sector [19,21]. Since then, school food is usually provided by local authority catering services or large commercial catering companies with a smaller proportion of schools using their own staff or small caterers. All children in the UK are offered a meal at lunchtime which is state funded for all children in the first 3 years of school and for disadvantaged children under the age of 16. Parents can choose to send their child with a packed lunch if they prefer. Primary school children are usually offered a full meal whereas secondary school children are usually offered a canteen style service. Procurement contracts for the supply of school meals are often put out for tender and awarded on criteria that are heavily biased towards the lowest cost provision to improve financial efficiency, leading to profit and competition being the dominant forces at the expense of other values such as dietary health and quality [20]. This may have had a substantial impact on school food quality since the devolution of responsibility from the local authorities as many kitchen facilities and skilled cooks who were capable of cooking from scratch with fresh ingredients were replaced with equipment and staff more suitable for reheating cheaper processed convenience foods [21–23].

Given the urgent need to address the impact of poor diet on population health in the UK and the ability of school meals to facilitate improvement, it is important to establish what is known about the contract types governing the provision of school meals. Understanding how these contracts are linked to the quality of food provided and outcomes for children will support further research to enable relevant and impactful policy and practice to be developed. This literature review sought to establish what is known about the types of contracts that exist for the procurement of school meals and to identify existing evidence that links these provision contract types with the quality of food provided and the key outcomes for school children. For the purpose of this study, provision of school meals refers to the offer of a full meal for a child on the school premises at lunchtime and key outcomes relate to child health and academic results.

## Methods

A scoping review was conducted to establish the breadth and extent of available evidence in this complex, but novel area [24–26]. A rigorous, reproducible, and transparent methodology was adopted based on appropriate guidance from The Cochrane Handbook, Joanna Briggs Institute (JBI) Manual and PRISMA-ScR checklist [24,25,27]. Criteria in these guidelines were addressed through the preeminent Arksey & O’Malley [28] framework as follows: Identifying the research question; Identifying the relevant studies; Study selection; Charting the data; and Collating, summarising, and reporting the results. This was supplemented with recommendations from Tricco et al., [29] enhancing the guidance on each of the individual framework stages, such as contextualising findings and presenting them in a clear and concise manner with key messages highlighted. Narrative synthesis techniques were used to identify themes which enabled a clearer presentation and credible discussion of the results [30].

### Literature search strategy and selection criteria

The search criteria were developed based on a standardised Population, Exposure, Outcome (PEO) framework to enable transparency and repeatability [31]. **Population** was searched using overarching terms of child and any extended variations, the institutional setting being school or education and the element being primary and secondary with alternatives of junior, infant, elementary and high. The **exposure** of interest (independent variable) was kept broad to capture the full range of procurement contract types for the provision of school food. Overarching terminology of purchase, procure, contract, and buy, along with the key components of catering and meal provision being lunch, dinner, and menu was used. **Outcomes** of interest (dependent variable) were based on factors which could link school food provision with child health and academic results. These were related to food, nutrition, diet, healthy, BMI, quality, uptake, absence, attainment, and results. Quality is often defined in different ways, for example, nutrient composition or meeting nutritional requirements, guidelines or standards. For the purpose of this study a sensitive approach was taken to include papers based on their own definition of quality. The review also sought to establish if further guidance was given in the literature for the quality of food in the context of school food provision contract types.

The following databases, being the preeminent databases for health and education research, were selected [24]: Medline; Web of Science; Scopus; Education Resources Information Center (ERIC). These were searched for peer reviewed articles and the reference lists of these articles were scanned for further applicable evidence. Google was used to search for grey literature. A new google profile was set up and location services were disabled to prevent results being influenced by previous searches or location [32]. Search terms (Supplementary Table 1) were inputted into the advanced search option using Boolean logic. The first 50 Google hits, being the 50 most relevant according to the search engine relevancy ranking, were reviewed for eligibility as is typical in systematic reviews [33]. Web pages for organisations involved in school food or public sector procurement are also a useful source of information relevant to this study; [34] therefore, the following websites were included in the search: Professional associations: LACA (the school food catering association) [35], Association for Public Service Excellence [36]; Government websites and agencies: GOV.UK [37], World Health Organisation [38]; Charities: The Food Foundation [39], School Food Plan [40]. The literature search was completed on 10 September 2023.

Criteria from the PEO model relevant to the review question and objective were used to determine eligibility of the studies [24,41–43]. All study designs, e.g., qualitative reviews, trials, and cohort studies were eligible for inclusion and scientific papers or letters, articles, reviews, book chapters or grey literature reports from reputable agencies and government departments were eligible. Conference abstracts, student dissertations or theses and protocol papers were not included. Documents dated from the devolution of school lunch procurement in 1988 to date of the search were included, as this is the point where the provision model changed in the UK. Eligibility was expanded to international studies to capture relevant findings from other countries however, only sources written in English were included, due to a lack of resources for translation. Any studies not relating to **populations** of primary or secondary school children were excluded (e.g., preschool children, colleges or universities, or adults). Any studies with **exposures** not relating to school lunch provision comprising of a full meal for the child or procurement contract type for the provision of that school lunch were excluded (e.g., purchasing by the student, home or family, packed lunches, beverages, and snacks, vending machines and tuck shops, marketing or advertising, food related education, physical activity, after school and breakfast clubs, childcare settings, or nutrition/ education interventions or studies). Any studies with **outcomes** not related to child health or academic achievement were excluded (e.g., addictive substances, child abuse, food allergies, COVID, food borne or other non-diet related diseases, eating behaviours or disorders, unrelated standard compliance, policies or guidance). Papers focused on global sustainability and food systems were excluded unless they also included research focused on areas that may relate to food quality. Operationally, this included those with data on food waste and organic/ local sourcing that also discussed food quality [44]. An iterative approach was used in case further applicable criteria became apparent during the review [45].

### Data extraction and analysis

Covidence systematic review software was used to ensure reliability, whereby the eligibility criteria at both the title and abstract stage were considered by a primary (NN) and experienced second reviewer (LP) to simultaneously review 10% of studies, which were selected using a random number generator. This process was repeated at the full text stage. At both stages, an inter-rater reliability score of 75% [26] was required to determine whether further papers needed to be reviewed or a third reviewer utilised.

Papers deemed potentially eligible after the title and abstract screen were read in full and data relevant to the research question were extracted and analysed using basic descriptive qualitative methods [42]. Whilst scoping reviews do not usually seek to synthesise the results of the existing evidence [28,42,43] a narrative synthesis was used to identify themes bringing homogeneity to the presentation of the results and to highlight the differences and similarities in the findings of the studies reviewed [46]. This approach has been particularly useful for synthesising different types of evidence in other studies [46–48] and was used to develop inductive descriptive themes in a methodologically rigorous way with reference to data returned alongside the study objectives and the research question [30,42]. Narrative synthesis as proposed by Popay et al., [30] was used to bring together findings and highlight key points.

## Results

### Literature search

**Fig 1:**
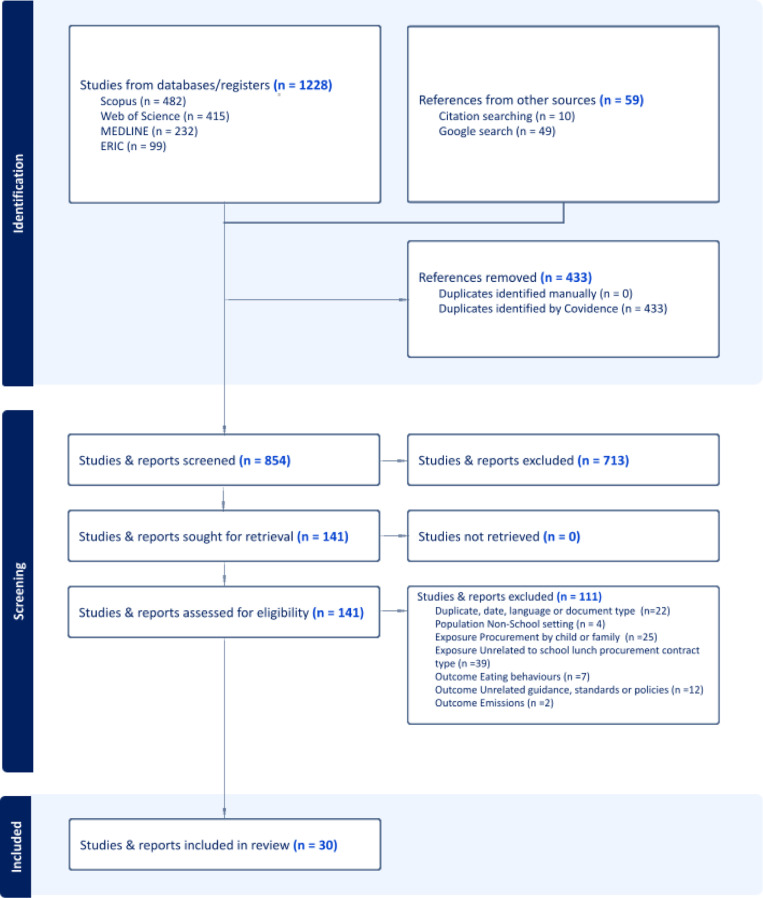
PRISMA flow chart of the literature selection process. PRISMA diagram showing the search and selection process for the scoping review.

The databases, citation review and google search returned 1,288 documents. After removing 433 duplicates, the titles and abstracts of 854 documents were screened for eligibility. This excluded 713 documents and the full texts of the remaining 141 documents were assessed for eligibility, identifying 30 papers and reports which fulfilled the eligibility criteria. Reasons for excluding documents in the full text screen are included in the PRISMA diagram (Fig 1). Over half of the papers (58%) were excluded for not meeting the exposure criteria, mainly due to focusing on procurement (purchases) by the student, or of food other than school lunch, or for examining education or nutrition interventions which were unrelated to school lunch provision or procurement contract type. A further 19% were excluded for non-eligible outcomes, such as eating behaviours or disorders or policies, standards or guidance notes unrelated to the specific outcomes of child health or academic achievement.

### Overview of included articles

The remaining 30 documents comprised 16 academic papers, 11 grey literature reports and 3 books (Table 1).

**Table 1:** Summary of documents included in review.

Document	Study Aims	Study Methodology	Population	Exposure	Outcome	Results
**Type** Author, Date, *Journal*	Aim/ objective as stated in the abstract	Overall approach to collecting the data	Sample size, Age, Ethnicity, Socio-economic status, **Location**	**Provision type**, Meal service, Financial & Contractual details	Outcome measured & method	Study findings, **themes** and relevant points
**Paper** Gonçalves et al., 2019 *Public health nutrition* [49]	To characterise the food environment in schools and identify individual contextual factors associated with hypertension and obesity.	Quantitative analysis of data collected in 2013/14 in the self-administered ERICA national school based survey.	Circa 73,000 adolescents aged 12-17 from 1,247 **Brazilian** public and private schools in 122 municipalities serving areas with> 100k inhabitants.	**Meals prepared on premises vs not** Contextual characteristics of school, e.g., location, administrative dependence, offer, sale and advertisement of foods School meal provision type.	Hypertension and obesity prevalence. Measurements: hypertensive = blood pressure ≥ 95^th^ percentile, obese = Z-score > 2 using BMI for age index.	School meals may be provided by the government in public schools or by commercial establishments. Characteristics of the school food environment (individual and contextual) were found to be associated with hypertension and obesity. • Ultra Processed Food was frequently available in the schools. • Almost all public schools in the study (97.9%) offered meals prepared on the premises in line with guidelines. • Commercialised foods for purchase in a cafeteria was predominant in private schools. Obesity prevalence was lower in schools where the meals were prepared on the premises vs not; 7.8%, (95%CI 7.3, 8.4), vs 11.0%, (95% CI 9.7, 12.3). Students had 35% lower odds of obesity in the schools offering prepared meals. Obesity prevalence was lower in schools with no commercialisation 7·3% (95% CI 6·6, 8·2) than in schools where foods were sold in the cafeteria 9·2% (95% CI 8·6, 9·9). Conversely, there was higher levels of hypertension where the meals were prepared in house 9.7% (95% CI 9.1, 10.5) vs 9.1% (95% CI 8.1,10.1). Individual characteristics were more influential than the school context on hypertension (86.2% vs 13.8%).
**Paper** Anderson et al., 2018 *Journal of public economics* [66]	To test whether offering healthier meals affects student achievement as measured by test scores.	Quantitative difference-in-differences style regressions using variation taking advantage of frequent meal-vendor contract turnover.	All **California USA** public schools over a five-year period public elementary, middle, and high schools with non-missing state test score data (approximately 9,700 schools across 900 districts)	**Healthy Eating Index (HEI) vendor vs not** Breakfast and lunch vendors at the school level classified as healthy or standard using a modified HEI. A vendor was classified as healthy if it’s HEI score was above the median score for all vendors in the sample.	School-by-grade-level standardized test results (STAR test)	Most schools had an in-house provision for school meals with only 12% provided through contracts with external vendors. Four different models of outsourcing supply exist • Private company provides the meals but school employees cook, handle and serve the food • Private company provides the food and the staff to cook, handle and serve it • Private company provides consultation services only • Another public school district provides the meals provision (this accounted for only 1%) Estimating the effect of healthy meal providers on **academic performance** is challenging due to difficulties in accurately measuring nutritional quality. However, students from schools with healthy meal vendors had slightly higher STAR test results (0.03 to 0.04 standard deviations) relative to in house provisions, especially among economically disadvantaged students. There was no impact on test scores from schools with standard vendors. Healthier meals did not increase meal uptake indicating that food quality, not quantity, improved academic results. No decrease in obesity rates was observed, possibly because all vendors adhered to the same calorie requirements.
**Paper** Chang 2014 *International Food and Agribusiness Management Review* [73]	To investigate the association between school lunch programs and children’s body weight	Quantitative data analysis of nationwide survey dataset (Nutrition and Health Survey in Taiwan for Elementary School Children (NAHSITC) using a mixed multinomial logit model	2,017 **Taiwan**ese elementary school children aged 6-12 in 102 schools	**Outsourced vs inhouse** School lunch provision No lunch Provider, In house, or Outsourced	Children’s BMI Data adjusted for time spent TV watching, eating breakfast, parental characteristics, home and school size	There are three different types of school meal programs for elementary schoolchildren in Taiwan: no school lunch programs; school lunch programs with meals from restaurants outside of school; school lunch programs with meals prepared in on-site kitchens at school. Children attending schools serving lunches prepared in house have lower weight on average (BMI, 16.18) and were less likely to be **overweight** (24.2%) than children attending schools purchasing lunch from outsourced caterers and restaurants (BMI, 19.29, overweight, 28.6%) or children bringing a packed lunch (BMI, 17.92, overweight 26.1%).
**Paper** Lassen et al., 2019 *International journal of environmental research and public health* [50]	To examine compliance with food service guidelines for hot meals as well as self-evaluated focus on food waste reduction	Cross sectional study using self-administered questionnaires. Data analysed using descriptive statistics and multiple logistic regression models.	680 **Danish** canteens in public and private elementary schools (enrolling children aged 5–16 years), public upper secondary schools (enrolling students aged 15–19 years) and public and private workplaces	**Outsourced vs inhouse** *Canteen characteristics* include the use of organic food, having a meal policy, number of daily lunch meals served, serving system and outsourced to external contractors vs. those operated by the workplace/school.	*Compliance with guidelines* Servings of fruit and vegetables, fish, wholegrain product and high fat meat and dairy products. *Food Waste Reduction* Self-assessed survey questions.	The inclusion of organic food was associated with meeting all 5 criteria, minimum fruit and vegetable content, and limiting high fat meat servings. Outsourcing to external contractors was associated with compliance of high fat meat and non whole grain product limits. There was no significant difference in **guideline compliance** between outsourced and in house canteens except for non whole grain products 0.50 (0.33, 0.75) P = 0.001. Most canteens focused on reducing **food waste** especially those using organic produce and having written meal policies.
**Paper** He et al., 2014 *Public health nutrition* [51]	To investigate whether public organic food procure- ment policies have the potential to induce changes in the school food service environment.	Comparative cross-national survey in 2009/10 using web-based questionnaire on attitudes, intentions and actions towards organic school food provision. Part of the ORE Research Pilot Project, innovative Public Organic food Procurement for Youth (iPOPY).	School food coordinators (SFCs) in public primary and/or secondary schools (children aged 6-15) in **Finland** (250 schools nationwide), **Germany** (122 schools from Hesse) and **Italy** (215 schools from eight provinces).	**Organic vs non organic** Organic sourcing policy aiming for defined proportion of organic ingredient in school meals.	Attitudes of SFC to: organic food and healthy eating; creation of an environment conducive to healthy eating; and how the school supports these attitudes and intentions.	Non-organic schools were 0.14 times less likely to adopt food and nutrition policies (P < 0.001), WHO health-promoting policies (P < 0.001) and have canteens (P = 0.017) than organic schools. Finnish schools and organic schools were most likely to have canteens (P = 0.001 and P = 0.017 respectively). Italian schools were most likely to serve nutritionally calculated meals (P < 0.001) and recommend healthier food choices (P < 0.001). No association was found between school type (organic/ non organic) and the existence of nutritionally calculated menus, or enforcement of nutritional recommendations. All SFCs supported **organic procurement** and promoting child health, regardless of the school type, but school food systems are managed at a higher level, limiting SFCs influence and ability to make changes. Quality of food is a high priority in Italy There is a gap in knowledge of effects of public organic food procurement policies on healthier school food environments.
**Paper** He et al., 2014 b *Perspectives in public health* [52]	To examine the possible influence of organic food sourcing policies on the development of healthier school food environments.	Cross-sectional quantitative analysis of data from a web-based questionnaire using the theory of planned behaviour model.	179 school food coordinators (SFCs) in 83 **Danish** public primary schools (6-15 years).	**Organic purchasing policy vs not** Presence of Public Organic Food Procurement (POP) policy committing to a proportion of organic content in school meals.	Attitudes, intentions/policies and actions in relation to organic and healthy foods served in the schools	Danish schools do not typically have a publicly organised food service and most children take a packed lunch to school.**Organic food policies** in schools may have potential to support a healthier school food environment. More organic schools had a food and nutrition policy (FNP) 80% vs 57% in non-organic schools and were more likely to apply nutritional recommendations indicating a prioritisation of healthy eating. More organic schools recommended healthier eating to their students than non-organic schools. However, there was little evidence that having an organic purchasing policy was directly related to healthy eating in the school. Very few differences in attitudes to healthy eating existed between different types of schools.
**Paper** Taylor et al., 2014 *Journal of public health management and practice* [65]	To describe environmental change strategies implemented to reduce sodium in school meals	Descriptive desk-based evaluation of the environmental change strategies	School administration and food service staff in 5 Anderson Union High School District schools in rural, northern **California USA**. Serving approximately two thousand 9th- to 12th-grade students.	**No comparison just inhouse** Public Health partnership to (1) facilitate changes to meal preparation practices, (2) improve cafeteria infrastructure,(3) provide training and assistance to improve procurement strategies.	Reduction in sodium in school meals	Onsite cafeterias in 2 schools also prepared meals for the remaining 3 schools. Kitchen equipment and staff skills supported the ‘heat and serve’ method where commercially produced food is reheated and served to students. Equipment was upgraded and training given to enable high volume, efficient, scratch cooking. Initial concerns of participants included higher costs and limited availability of low sodium ingredients and resistance to change from food service employees. Training and education on nutrition, health effects of high sodium diets and scratch cooking were given. Purchasing co-operatives were used to increase purchase volumes to lower prices and increase availability of low sodium items. The strategies to **reduce sodium** in school meals in 2011 led to an implementation of scratch cooking and improved procurement strategies which successfully reduced sodium levels in school meals.
**Paper** Shahril, et al., 2000 *Malaysian Journal of Nutrition* [72]	To evaluate the implementation of the School Supplement Feeding Program (SSFP)	Cross-sectional study, part of a larger study to assesses the implementation of SSFP and nutritional status of supplemented school children using a questionnaire and qualitative interviews with the school leadership and food operator	Primary school children (7-12 years), parents, headmasters/mistresses, heads of the school committees, teachers and food operators at 129 schools (77 national type, 31 Chinese and 21 Tamil vernacular) in four different regions (northern, eastern, central and southern) of Peninsular **Malaysia**	**Contractor vs local community** SSFP Food from 10 recommended menus prepared by (1) contracted operations (local community members) and (2) voluntary operations (including teachers)	Meal quality measured by nutrient content from sample meals compared to Malaysian recommended daily intake Financial management and budget disbursement Food preparation and menu selection	Most schools (84.5%) used a contract canteen operator with 1-2 year contracts. Others used local food caterers, and one school had teachers prepare the food. **Food quality** was better with voluntary staff or food caterers due to lower operating and overhead costs. Nutrient content was proportional to the budget per child with more expensive menus having considerably higher nutrient content. Some menus did not meet recommendations for certain nutrients, possibly due to cooking and preparation methods. Operators stayed within budget by mixing high and low-cost menus and sometimes served non-recommended foods preferred by the children. Slow disbursement of funds to schools caused financial instability affecting operators ability to meet recommended quality standards.
**Paper** Chiaverina et al., 2023 *Journal of Agricultural & Food Industrial Organization* [53]	To assess the nutritional quality and the carbon footprint of school menus. Including the relative merits of: (1) direct management by municipalities (in house provision) (2) outsourcing the catering service (delegated provision)	Literature review on school canteen meal nutritional quality and carbon footprint. Review of primary school menus downloaded from websites	Primary schools in 101 **Paris**ian inner suburban municipalities	**Outsourced vs inhouse** Management mode Socio economic and political orientation of municipality Education level and canteen size	Nutritional and environmental quality of school meals. Nutritional quality measured through menu compliance with 2011 national guidelines: (1) for the whole criteria (15FC) (2) for the four most nutritionally important criteria (4FC)	Comparisons of the inhouse model of meal provision to a delegated (to private contractors) model showed mixed results. Poor **quality** arose from contractual ambiguity and unsuitable selection processes in meals from private caterers and a lack of resource, nutrition expertise and administrative inflexibility in house providers. Lower quality found in outsourced canteens was potentially attributed to predatory pricing strategies to win contracts on an unsustainable cost base, contractual complexities and higher management costs leading to reducing levels of the more expensive healthy foods such as fruit and vegetables. In house canteens scored significantly better for nutritional quality on the 15FC (mean 12.99 vs 12.22) but not on the 4FC (mean 3.52 vs 3.57). Larger facilities had better capacity to provide healthier menus with economies of scale enabling better management and nutrition resources. Higher levels of organic produce could lead to higher nutritional quality due to the additional training needed to implement more creative menus to offset the cost of organic produce.
**Paper** Hill et al., 2023. *Public health nutrition* [74]	To analyse the healthiness and price of items available in schools and associations with school characteristics.	Quantitative analysis of a cross sectional sample of canteen menus using descriptive statistics and regressions	48 primary (elementary) schools in Victoria **Australia**	**Outsourced vs inhouse** Collected characteristics: (1) remoteness; (2) sector (government or Catholic/independent); (3) type (prep to year 6 (aged 5–12 years) or combined prep to year 12 (aged 5–18 years); (4) size; (5) socio-economic status.	Adherence to government guidelines for provision of food and drinks in primary school canteens. Dieticians classified individual menu item as ‘green’, ‘amber’, ‘red’ or ‘black’ based on its nutritional content, using the state policy traffic light system	In Australia school lunches are typically brought from home or purchased from a school canteen which may prepare the food in house or outsource provision to a commercial provider. No evidence of association between school characteristics and **healthiness** or **pricing** of food except for regional centres which had the highest proportion of (black) banned items. Unhealthy items were cheaper than healthy alternatives. Compliance with state menu guidelines was low. The food service policy for schools is not formally monitored and it is not mandatory for non-government or independent schools
**Paper** Galli et al., 2014. *Sustainability: Science Practice and Policy* [54]	To understand the reciprocal relationship between professionals and users of school meal services as a driver to mobilize new resources that steer service innovation and a shift towards more sustainable practices.	Case study with exploratory semi-structured interviews drawing from the theory of co-production. The collection of general information on the service through access to relevant documents and on the projects being developed	Pisa **Italy** The main stakeholders involved in the school meal service (The Canteen Committee)	**No comparison** School meal service participation	Sustainability and child health	Most (74%) Italian municipalities subcontract school meals to caterers with their own structures and staff, 15% are provided by public administration with full control over facilities and staff and 11% are a mixture of the two models. National guidelines for school catering services include guidance on technical specifications for tendering, procurement to support a variety of seasonal, sustainable, ethical and fresh ingredients to improve nutritional quality. Quality is monitored to reduce waste particularly of fish and vegetables and local farmer contracts support the local economy and improve quality. A5-year, €16m contract is awarded based on service (65%) price (35%) and quality (5%). Two thirds of meals are prepared off site and transported to the schools. Trust and working relationships can be impacted by the trade-off between price and quality of the service.
**Paper** Lavall et al. 2020 *Nutrition* [55]	To perform a nutritional assessment of the menus served in school can- teens and to verify their effects on the nutrition of schoolchildren.	Quantitative descriptive statistical analysis of nutrient content of menus.	4 public schools with different management models as well as different supply patterns 3 collective catering companies offering ~ 53 500 menus per day in 369 schools in Valencia **Spain**	School kitchen vs company kitchen (i.e., **Outsourced vs inhouse**) Catering contract type menus	Nutrient content of food 15 samples analysed in laboratory using common nutrient extraction methods	There are several contract types for school food provision in Spain with a trend for outsourcing to external companies either preparing the food in its own kitchen (63.6%) or with a kitchen in the school (36.4%) There was a large variation in calorific content between schools and between days. **Compliance** with recommendations varied with all schools providing adequate protein but only 3 schools meeting carbohydrate recommendations. Energy intake from fats was higher than recommendation in 2 schools, lower in 1 school, and compliant in the other. In all cases sodium recommendations were exceeded, with the highest levels in menus with precooked dishes and processed meat.
**Paper** Tregear et al. 2022 *Journal of cleaner production* [56]	To measure and compare the environmental, economic, and nutritional outcomes of different models of school meals procurement.	In depth Case studies capturing different procurement model types. Qualitative in-depth interviews and quantitative analysis of nutritional meal composition, economic impact and carbon footprint.	Ten primary school catering services in **Croatia, Greece, Italy, Serbia, UK**	**Low-cost model (LOW) or local sourcing model (LOC)** or in the case of Italy organic model and local organic model	Nutrient content from a sample of 20 menus across 2 seasons using the national food composition database for each country and evaluated against national nutrition standards for primary school after adjustment for plate waste	Primary focus was on **sustainability** but 1 of 3 aims also addressed **nutrition** No consistent patterns were identified linking the procurement model and the nutritional quality of the menus. Rather, the nutritional quality of the menus was driven by robust adequately resourced implementation of nutritional standards with the help of qualified nutritionists. Croatia: national nutrition standards exist, schools manage contracts except for core items like milk and bread arranged by the council. Meals are usually cooked on site at €1.20 each. Greece: state funded meals at €2.22 each. Municipalities contract private firms to prepare and transport meals. No national nutrition guidelines. Italy: high quality meals and nutritional standards with regional legislation supporting local, organic products. Meals are organised municipally often contracted to private firms for preparation and transportation. Prices in case studies were €6.18 and €5. Serbia: national nutrition standards exist. Procurement polices favour the lowest cost tender. Meals are organised at school level, often outsourced to private companies. Prices range from €1.02 to €1.21 UK: case studies show meals either contracted to private caterers preparing meals on site at €2.28 or by local authorities cooked on site for €2.21 to €2.27. Results indicated suboptimal nutrition from menus. Plate waste was high (30%), mostly fruit, vegetables and starchy foods affecting fibre, energy and carbohydrate intake. The most nutritious Italian menus generated the most plate waste (38%) while Croatian menus with the most nutrient noncompliance had the least plate waste (12%),indicating that nutritionally balanced meals may not be appealing to the children.
**Paper** Brinck et al., 2011 *Perspectives in public health* [57]	To present a government-planned intervention concept of 40 days of free school meals intended to kick- start the implementation of healthy school meal systems in Danish schools.	Semi structured qualitative interviews based on a phenomenological-hermeneutical design seeking the stakeholders’ own understanding with analysis based on implementation theory and the construction of a program theory.	35 headmasters and 5 school meal suppliers in 35 **Danish** schools	**No comparison** Intervention to provide funding for 40 days free school meals for all pupils	Establishment of a healthy school meal system	A lunch bag provided from home and eaten in the classroom is the usual school lunch provision in Denmark and canteen facilities are rare. Most schools (33) chose an external provider with limited involvement of the schools and only 2 chose to produce them in house. After the free provision ended take up fell to unsustainable levels and many commercial suppliers withdrew services. Suppliers cited a lack of experience in school food provision as an issue and headteachers mentioned the lack of flexibility of and accessibility of the web-based ordering systems. Other factors were a lack of: stakeholder involvement;, diversity of meal choice; support materials; kitchen facilities and skills in the schools; and a lack financial viability for the provider.
**Paper** Lawrence and Liquori 2012 *Childhood obesity* [67]	Describes key dimensions of the work of collaborative network School Food FOCUS to support procurement change towards cost effective, healthful and sustainably produced school food.	N/A	**USA**	**No comparison**	N/A	The procurement infrastructure is now favours large national commercial organisations making it expensive and complex to revert to smaller producers. Increased use of processed fast food is driven by convenience and **price** sensitivity with public institutions now operating a heat and serve model rather than cooking from scratch. Defining **food quality** is challenging due to the complexity of school food services and differing opinions on what constitutes ‘better’ food, influenced by nutrient content, production processes, economic, environmental and social justice goals, personal and cultural influences, and marketing messages from the food industry. FOCUS defined serving better food in schools as: • More healthful, based on meal pattern guidance; • More regionally sourced; • More sustainably produced Key challenges in providing quality food inschools: • Only $1 to spend on ingredients; • School food expected to contribute to budgets rather than be funded; • Complexity of policies and regulation; • Academic focus reducing lunch periods to less than 20 minutes.
**Paper** Mikkelsen et al., 2005 *Food Service Technology* [58]	To present and discuss the findings of the European Network of Health Promoting Schools group and its implications for school food service in the future; to discuss how schools can become a more active arena for the promotion of healthy eating, and how food service can contribute in this respect	Forum of nutrition experts to discuss the results of a survey exploring the provision of food in schools across Europe, to find out how food provision is linked with nutrition education in primary and secondary schools and to study the extent to which the provision of food and nutrition education are embedded in the Whole School Approach.	**15 European countries**	**No comparison**	N/A	Editorial review noting ‘considerable diversity’ in the organisation, operation, and finance of school food provision across participating countries. Close co-operation between stakeholders is a key factor of success. ‘It is not the responsibility of the food services operator alone to take responsibility for **healthy eating** in school’ In most countries schools organise their own food provision. Some have clear national policies, others delegate to regional or local authorities some have no tradition of providing school meals Primary schools typically offer traditional meals or packed lunches. Secondary schools have varied options, including traditional meals, packed lunches, vending machines and cash cafeterias. In cultures like Spain and Belgium, children go home for lunch. Countries like England, Finland, France, Scotland, and Sweden provide hot meals through school or outsourced caterers. The **financing models** vary with some countries subsidising meals for all children and others for specific groups such as socio economically disadvantaged children. Subsidies can be national, regional, local, or school provided. Adequate pricing is needed to cover costs and avoid selling unhealthy but profitable products to maintain financial viability.
**Grey Literature Report** Rylander 1999 *Food for Thought: Ideas for Improving School Food Service Operations.* Texas State Comptroller of Public Accounts, Austin [68]	Shared examples from school reviews “to reduce cost of auxiliary services, like food services… to channel more education dollars to the classroom, where it belongs”	N/A	Texas **USA**	**Outsourced vs inhouse** N/A	N/A	Guide for Texas school districts from Comptroller of public accounts focused on **cost savings** for school meal provision. • Check if outsourcing provides better service at lower cost, with regular evaluation, monitoring, and contractor accountability • Include mutually agreed performance standards in contracts to incentivise good performance such as improving quality and controlling cost and to penalize poor performance. • Conduct tough contract negotiations and thoroughly review terms and conditions before renewal considering costs of conducting those same services in-house. • Privatisation can reduce costs, and improve uptake quality, service, customer satisfaction and employee care.
**Grey literature Report** Nicholas et al., 2006 *Review of the school meals service and other school nutritional issues in Wales* National Foundation For Educational Research [59]	To gather evidence on Local Authority and school approaches to nutrition in schools to inform the work of the Welsh Assembly Government Schools Food Task and Finish Group.	Mixed methods including literature review of recent legislation and published research on nutrition in schools, two questionnaire surveys to gather quantitative data and qualitative interviews with a range of stakeholders.	**Wales** Qualitative interviews with key personnel in 8 Local Authority (LA) school meal service and 9 schools’ staff and students. Survey questionnaire from 10 Welsh LAs and 79 schools.	**No comparison LA only**	N/A	• Service team **quality** and their understanding of the issues are key factors for school meal provision. • Various procurement methods and selection criteria exist including LA procurement collaborations. • Tendering processes are common to assess cost, quality, and other criteria such as service, sustainability, local produce, capacity and traceability. Cost:quality ratio varied from 70:30 to 40:60 with a shift towards quality. • Levels and methods of quality control differed between LAs with some relying on school staff and environmental health departments. • The service is under funded impacted by low-cost strategies following compulsory competitive tendering. • To remain competitive LAs reduced costs by reheating and serving processed food instead of scratch cooking, lowering quality and meal uptake. • Primary schools meal prices ranged from £1.40 to £1.65 with less than 40p spent on the food. 70% of LAs make a loss subsidising school meals from other budgets such as education. Pressure for profitability influences policy decisions. • Choice and food quality influence uptake but results and views on quality were mixed. • On-site food preparation is perceived better quality, but funding and facilities are lacking. • Other-quality determinants include outsourcing to private providers under PFI agreements, staff recruitment and retention issues, and inadequate nutritional standards, legislation and funding.
**Grey Literature Report** Robinson 1996 *School Lunch Program: Role and Impacts of Private Food Service Companies*. United States General Accounting Office [69]	To examine (1) extent to which schools use private companies to operate their lunch program and the impact on the National School Lunch Program; (2) terms and conditions in contracts between schools and food service companies	Quantitative data from questionnaires and analysis of a random sample of 68 contracts.	1,175 **USA** food authorities contracting with Private Food Service Companies (FSMC) 765 US food authorities not contracting with FSMC 1,887 US public school cafeteria managers	**No comparison FSMC only**	N/A	US General Accounting Office (USGAO) report to congressional committees • Outsourcing was mainly for **financial** reasons, aiming to increase revenues and cost effectiveness, reduce administrative burdens, increase uptake and improve **nutritional value** of meals. • Costs and deficits were reduced and take up increased with FSMC but was not better than non-outsourcing authorities. • Fee structures varied, mostly cost-plus fixed fee annually or per meal. • Many contracts did not comply with the federal requirements, particularly in retaining control over meal services.. • Schools using FSMC were more likely to offer brand name fast foods, such as pizza, subs and burritos, increasing from 2% to 13% in 5 years. The main reason was to increase uptake, while non-offering schools cited nutritional quality.
**Grey Literature Report** Williams et al., 2021 *Study of School Food Authority Procurement Practices*. U.S. Department of Agriculture, Food and Nutrition Service [70]	To identify and describe SFA contractual practices with FSMCs	Quantitative data from a web-based survey and qualitative interviews with a subset of survey respondents.	Nationally representative sample of **USA** School Food Authorities (SFA)	**No comparison FSMC only**	N/A	Report for US Department of Agriculture, Food and Nutrition • **Cost** (31.4%) was the main factor driving contract type followed by service **quality** (21.2%) and product consistency (17.3%). Fixed price contracts were selected in 57.6% SFAs for cost control. Cost was the main factor influencing procurement method (63.1%) quality did not feature on the list. Tender documents included specifications for quality, serving size, volume, and nutritional content, with 68.5% monitoring contractual performance. • 26.2% of SFAs use FSMCs, with 51% managing all procurement and 37% managing some aspects. Only 52.5% examined FSMC management of quality goods/services and 18.6% did not know how FSMC performance was monitored, if at all. • Small SFAs used FSMCs more due to the reduced administrative burden, cost and difficulties meeting nutrition guidelines. • Reasons for using FSMC included; procurement compliance (57.1%); delivery management (49.8%); coordinating all procurement (48.7%); procures products (48.5%); lower prices (42.9%);consistent prices (41.4%); year round product availability (38.8%); past use (38.5%); hiring staff (38%); FSMC already in place (32.7%) • 10 SFAs reported issues with communication, staff management, food quality and menus.
**Grey Literature Report** Hacking 2022 *APPG on school food report - impact of food cost on school meals*. Association for Public Sector Excellence[60]	To establish how the cost-of-living crisis and rising cost of food was impacting school food.	Quantitative descriptive statistics and narrative summary of data from an online survey	**England** Survey responses: local authority caterers (21.98%) school caterers (19,23%) parents (15.38%) school staff, (13.74%) private sector caterers (8.79%) charity/ not for profit organisation (7.14%) frontline catering staff (1.65%) school governors (1.10%) School Business Managers, school food suppliers, grandparents, trade unions and other local authority officials (10.99%)	**No comparison caterers as a whole**	N/A	Report for All Party Parliamentary Group on School Food The rising **cost** of providing a school meal has made the school meal service increasingly unsustainable. With 10% of caterers reporting reducing **quality**. Impact of cost pressures on quality and menus: • Long term contracts with caterers means complaints are not dealt with and profit prioritised over child wellbeing; • Time saving food prioritised over nutritious food; • Processed food and packet mixes used more regularly; • School food standards not being fulfilled; • Portion sizes reduced and expensive healthy food limited; • Difficulties creating dishes on budget; • Protein and fish being reduced/ removed; • Variety reduced and menus simplified; • Debt rising; • Food cost per meal per day rising from 88p to 95p; • Catering company cancelled financially unviable contract in small rural school and other catering companies would not tender; • Underfunding pushing catering companies away from the market and families towards unhealthier packed lunches; • UIFSM and other budgets being used to fund the deficit in school meal provision.
**Grey Literature Report** Dimbleby and Vincent 2013 *School Food Plan & Evidence Pack* [19]	Recommendations and action plan to transform the English school food service to provide tasty nutritious food available to all children. Data pack to support the development of the school food plan	Independent Report for the Department of Education to recommend what the government can do to get children to eat well at school. Detail of analysis by consultants OC&C to support development of the school food plan.	**England**	N/A	N/A	Overall, 32% of meals were private catered, 56% LA catered and 12% school catered. School meal provision is complex and varies widely by region • Getting the contract right is crucial;serving notice on a substandard contract takes specialist legal knowledge and can incur penalties, a good contract can increase profitability and **quality.** • In house services are not more expensive than external caterers with similar average costs. There was no evidence that academies taking catering in house without the mandatory compliance with school food standards had impacted the quality of the food. • The head teacher’s attitude is key to improving school food culture regardless of the provision contract type. • School lunch prices (£1.93 primary, £2.03 secondary) do not cover production costs (£2.30 primary, £2.41 secondary) with a 10% deficit covered by school or LA budgets. Provision contract type did not determine the costs, costs of in house provision were similar to LAs despite lower volumes. There were no comparable figures for private caterers. • The removal LA funding allocation has reduced their involvement in school food, impacting financial viability particularly for smaller schools
**Grey Literature Report** Soil Association. 2003 *Food for Life: Healthy, organic, local school meals* [23]	Review of the school food provision and recommendations for government to improve the quality of school meals	N/A	**United Kingdom**	**No comparison caterers as a whole**	N/A	Advocacy report from the Soil Association and Organix Brands plc • Poor **quality** school meals raise concerns about child health and low service uptake. Meals mainly consist of low-quality processed foods. Skilled cooks are replaced by low paid ‘food service operatives’ to reheat food with unknown provenance using sauce powders, cake mixes and frozen food. • LA catering services spend approximately 35p per meal on ingredients whereas private companies spend as little as 31p per meal to make a profit. • Contract caterers offered meals compliant with standards, but also served fast food options. Healthier diets were not prioritised with cheaper, less healthy alternatives enabling caterers to balance the consumption in their favour. • Private caterers’ cherry-picking lucrative contracts made some LA services unviable. Private sector catering contract details are difficult to access for schools to manage to. • In house food provision could improve quality and uptake.
**Grey Literature Report** Expert Panel 2002 *Hungry For Success: A whole school approach to school meals in Scotland* [61]	Expert panel on school meals recommending the introduction of monitored nutrient based standards supported by guidance to caterers	N/A	**Scotland**	**No comparison caterers as a whole**	N/A	Provision methods and facilities vary between LAs and schools with some preparing hot meals in house and others using central kitchens. Meal **price** and subsidy levels also vary between LAs Partnership approaches with a whole school approach to food supported by the leadership and stakeholders is recommended. Key factors influencing uptake are **quality**, choice, queuing, peer group choices, and food environment. Good practice excludes obvious commercial branding for school meals. Changes need an environment free from ‘competitive commercial pressures’
**Grey Literature Report** Food Matters. 2014 *School Food Matters Evaluation report: primary school meals improvement campaign in Richmond – 2007 to 2011* [62]	Evaluation of the effectiveness of the 2007 to 2011 school food matters campaign	Qualitative interviews, workshops and documentary analysis and quantitative data analysis	29 primary schools in Richmond **England**	N/A	N/A	Food matters is a not-for-profit advocacy organisation focusing on creating **sustainable** and fair food systems. Appointing a catering contractor committed to staff training and an improved food environment was key to success. Changing contractor was influential on the improvement of the **quality** of meals. Contract specification of best value • was not clearly defined allowing caterers to interpret them to their advantage. • aim was to provide high quality nutritious catering service but focus was on minimum acceptable standards • no specification regarding where the meal was to be prepared allowed meals to be prepared in Wales, frozen, and then distributed via a hub to schools where they were reheated. • often interpreted as lowest **cost** leading to low quality, poor uptake, and a financially unviable operation. A contract precisely specifying food quality, nutrition, and ingredient sourcing requirements without the ability for open interpretation by the contractor led to improved facilities, staff training and motivation, better quality and lower prices.
**Grey Literature Report** RSM Ireland 2022 *DEPARTMENT OF SOCIAL PROTECTION Evaluation of the School Meals Programme* [63]	Independent evaluation of school meals programme by RSM Ireland for the Department of social protection	Surveys, interviews, focus groups and workshops with stakeholders across the school meals programme using a theory of change approach	**Ireland**	**No comparison caterers as a whole**	N/A	Irish schools choose their meals provider on the open market and the contractual relationship is between the school and the caterer. The process is governed by public procurement rules and compliance with nutritional standards but there is little if any practical state involvement in the process. Most schools do not have cooking facilities or canteens and most food is delivered in. Characteristics of the model: • Increased concerns regarding **waste** generated from packaging and uneaten food. • The freedom to choose their model of provision facilitated innovation • Wide variation in the experience and provision of the school meal service. • Investment in kitchen facilities was difficult for contracts less than 5 years as it was not enough time to pay back the capital commitment.. Recommendations • A new system of procurement to enable better control over the tendering process and to establish a structure to monitor and evaluate quality and value for money of the meals provided. • Realistic pricing to provide quality school food whilst remaining a financially viable business. Additional compensation required for more expensive provisions such as small rural schools. • Mandatory open book accounting to prevent excess profits being made by corporate providers at the expense of the public purse and to ensure that they receive adequate economic returns to provide a quality provision for the lifetime of the contract.
**Grey Literature Report** Dimbleby 2021 *National Food Strategy: The Plan* [3]	To understand how the food system works for population and planet health and preventative interventions.	Independent review of the food system for the UK government.	**England and the United Kingdom**	**No comparison caterers as a whole**	N/A	Just four contract caterers hold 61% of the public sector market. This reduces the power of the state to drive high **quality** and further competition in the market is needed to increase standards. The current tender evaluation process has a 50% to 80% rating based on price which does not facilitate the provision of high-quality food. School children are highly dependent on publicly procured food with up to half of their daily food coming from school and for some children representing their only substantial meal of the day.
**Book Chapter** Ebdon and Chen 2017 *The Intersection of Food and Public Health: Current Policy Challenges and Solutions*. Taylor and Francis [71]	To examine the impact of the privatisation of school food services on school food cost and quality. To explore the rationale for, and the benefits and challenges of, contracting vs internal school food service provision	Exploratory anonymous qualitative interviews with school district and private food service company managers and documentary review of financial statements, procurement policies and contracts.	15 School districts in Nebraska (7) and Florida (8) **USA** Small sample size which may not be generalisable	**Outsourced vs In house** Food service provision type in house (8) or contracted out (7)	(1) Reasons for contracting (2) Contracting vs internal operations (3) Contracting process and terms (4) Effects of contracting	Evidence for cost savings from the privatisation of public services is inconsistent and perceived levels of competition and associated cost savings do not always materialise. Privatisation is criticised for lack of transparency and accountability, reduced flexibility and control and lower **quality**. Both contracted and internal provisions had difficulty with uptake of healthy meals due to student preferences for fast food. Features of Contracting: Full responsibility for operational service and compliance; Financial efficiency, purchasing power and economies of scale; Resources and expertise (e.g., nutritionists); Professional management to reduce costs and manage staff; Often purchase nationally impacting local community and economy; Complex contracting and transition process; Contractors may not ‘keep their promises’, re tendering process is time consuming and costly; Management fees can increase costs; Misaligned goals, contractor focuses on profit and school on education and well being. Features of Internal operation: Retain control over menus and operation; Alignment goals with long term education and student needs; Supports longer term investment decisions; Connection between food and education; Personalised service from a better knowledge of the school, community and students; Can be cheaper with a good internal director. Contract types identified were fixed price (n = 5) or cost reimbursement (n = 2) and ranged in length from 1 year to over 20 years.
**Book** Morgan, and Sonnino2008 *The school food revolution: public food and the challenge of sustainable development*. London: Earthscan [64]	To explain why the locally sourced school meal, such a simple confection in theory, turns out to be surprisingly complex in practice	Case studies of school meal provision	Schools, governments, and meal providers in **New York (NYC), Rome, London, South Gloucestershire, Carmarthenshire and East Ayrshire.**	Not stated but includes historical context, policies, legislation, and culture	**No comparison caterers as a whole** Sustainable development through the provision of locally sourced ingredients (operationalised through 3 principles of economic development, democracy and environmental integration required to sustain a food system over time and how a carefully planned and managed food system can impact the implementation of those 3 principles)	In the US and UK. A 4-5 yearly retendering cycle for school meals focuses on reducing prices and therefore quality. Additional nutritional standards complicate the process. Financial viability depends on uptake, with many UK local authority caterers breaking even (42%) or making a loss (51%). Rising operating costs and falling uptake make the service non-viable for many private contractors. Public sector procurement officials can lack the business experience and understanding to manage and design tenders, negotiate favourable terms and conditions with large private sector suppliers. In Rome external contractors manage most services, with tenders designed by LAs and supported by the central department of education. Essential service quality criteria requires mainly fresh organic fruit and vegetables. Award criteria is based on price (51%), service (30%) and environmental criteria (15%) with the remainder for food education and interventions. Officials worked with caterers to make meals financially viable at €4.23 euros per meal. There is very strict compliance monitoring in every school. Good practices exist in both private and LA provisions but effective change is more likely where the public service is good as it can reach a greater number of schools. A lack of LA control and influence hinders refom. Cultural differences impact food quality philosophy, with the UK’s cost based culture contrasting Italy’s strong food culture. UK and US caterers compete with cheaper, and potentially more appealing, junk food unlike Italy, Finland and Sweden where school meals are seen as a public investment in health. NYC and Greenwich UK serve healthy food styled like fast food emphasizing branding and marketing in a dining hall looking like a franchise restaurant rather than an institutional feeding centre.
**Book** Swensson et al., 2021 *Public Food Procurement For Sustainable Food Systems And Healthy Diets* [20]	To provide evidence on sustainable food systems and support the practical implementation of sustainable public food procurement initiatives.	Systematic literature review and case studies on the contribution of public food procurement to food and nutrition security	**North and South America, Europe, Asia, and Africa**	**No comparison caterers as a whole**	N/A	Food and Agriculture Organisation of the United Nations (FAO) publication focused mainly on **sustainability** and other public sector procurement principles unrelated to school food contract type. The length of public procurement contracts and monitoring systems are important for the stability and nutrition outcomes. Catering services are concerned with profitability and may not be convinced that there is a business case for reducing food loss and waste if sustainable development goal (SDG) principles are not contractually binding and monitored or evaluated. The relationship with suppliers and control over food provision is delegated to the catering services company and the school or local authority will have less, if any, ability to align this with goals unless they are important to the contractor or specified contractually.

There was international coverage in the papers, and books from South America (n = 2) [20,49], Europe (n = 19) [3,19,20,23,50–64], North America (n = 9) [20,64–71], Asia (n = 3) [20,72,73] and Australia (n = 1) [74]. However, the grey literature related exclusively to the USA, UK, and Ireland.

The dates of the academic papers covered 23 years from 2000 to 2023 with the majority (75%, n = 12) [49–56,65,66,73,74] in the last decade. The grey literature and books were dated between 1996 and 2022 with half (50%, n = 7) in the last decade [3,20,60,62,63,70,71].

Sustainability, relating to organic procurement and waste, was a key focus of seven of the papers [50–54,56,67], ten were mainly concerned with nutrition or guideline compliance [50,53,55–58,65,67,72,74], two with body weight and disease [49,73] and one with academic achievement [66]. Different types of food provision contract types were described (see Table 1): outsourced vs in-house (n = 5) [50,53,55,73,74]; on premises meal preparation vs off premises (n = 1) [49]; organic vs non organic (n = 2) [51,52]; contractor vs local community (n = 1) [72]; healthy vendor vs not (n = 1) [66]; low cost vs local sourcing (n = 1) [56]. Five papers gave background information only and did not compare outcomes by provision contract type [54,57,58,65,67]. The academic papers were not necessarily focused on the comparison of health outcomes by procurement contract type, though the outcomes and procurement contract type were described and therefore the papers were included as eligible. One paper did focus on a comparison but framed it as low-cost vs local sourcing rather than provider contract type [56]. Including these papers captured important characteristics of each contract type providing context and understanding of how the contract type links with food quality and outcomes.

The 11 grey literature reports were mainly commissioned and funded by government departments or advocacy organisations to evaluate or inform policy. Therefore, they were more concerned with providing overall economic, planetary and population health related insight, as opposed to comparing specific outcomes by provider types. Only one report made comparisons between outsourced and in-house provision [68]. Two looked at food service management companies [69,70] and one at local authorities [59]. Two of the three books were mainly concerned with sustainable school meal provision and did not compare different provision contract types [20,64]; one specifically looked at outsourcing the provision and made comparisons to in-house provision [71].

### School meal provision contract types

Documents revealed a variety of school food provision contract types between and within different geographical areas. These fell into broad overall categories of government provision, in house catering and outsourced commercial provision. This varied by school type, geographical area, or political orientation [19,49,53,58,64,66,69,71]. In most European countries, the school was responsible for choosing the food provision contract type [58]; however, European school food systems were found to be commonly managed at a higher level than the school which could be at a national or regional level of government [51,54,56]. Countries such as England, Finland, France, Scotland, Italy and Sweden provided a hot lunchtime meal, either through the school, local government or an outsourced catering contract [54,56,58,64]. School meals were prepared in different locations, with some cooked on site and others prepared in central kitchens for transportation to the schools which could be by outsourced catering teams or in house staff or volunteers [54,56,72].

Differences also existed within provision contract types. For example, one US study found several variations of outsourcing school meal provision such as, private company providing the meals, but the school employs the staff; private company providing the meals and the staff; private company provides consultation services only; and on rare occasions (1%) another public school district provides the meals [66]. There was no peer reviewed scientific research on the provision contract types in England. The School Food Plan [19] provided the most comprehensive picture of provision; however, this report is now over a decade old. The report noted the complexity of school meal provision. This could be contracted by the local authority but provided in house through local authority catering services or through a private caterer. Alternatively, it could be contracted through the school which may use the local authority, a private caterer, or cater in house.

Identified papers indicated that good practice existed across the different provision contract types regardless of whether provision was executed by a private caterer, in-house staff, or a local authority catering service. The synthesis of findings highlighted a number of key themes linking the school food provision contract type with food quality and outcomes for children including; contractual relationships, economic factors, nutritional quality, disease, culture, academic achievement, policies and regulation and child acceptability.

### Contractual relationships

The importance of the contractual relationship and having binding, monitored, and evaluated specifications was noted to help to manage a complex situation where quality and outcome priorities may differ by procurement contract type. For example, there could be conflicting priorities of profitability for private contractors compared to social, economic, and environmental principles for authorities [20,54]. A lack of precision in contractual terms such as ‘best value’ had allowed interpretation by caterers to suit their business goals rather than the stated aim of a high quality nutritious catering provision [62]. Substandard contracts without legally binding detailed specifications were thought to impact school food take up, profitability, food quality and prices and expensive specialist legal knowledge would be required to renegotiate or exit the contract [19]. Tender design and contract negotiation were identified as key specialist skills which public sector officials and school leaders may not have despite their responsibility for the contractual relationships with large public sector suppliers [64]. This could affect quality through outsourced contracts being awarded at financially unviable rates, or too much (or too little) market competition driving flawed costing models [53,71]. Differing views on contract lengths were presented, with long term contracts thought to facilitate contractor complacency and profit prioritisation over quality [60] and conversely to facilitate capital investment in facilities which is not financially viable over shorter contract terms [63].

### Economic factors

The low levels of available spend for ingredients and the impact **financial viability** had on nutritional quality was highlighted in several reports [19,23,53,58–60,64,67,72]. Nutritional quality of food was related to the available budget, with convenience and price sensitivity driving the increased use of cheaper processed fast food over more expensive fresh food and the expense of reverting to an infrastructure supporting scratch cooking [53,67,72,74]. Examples of spending varied widely and ranged between 32p and £1.10 per meal served [19,23,59,64,67]. Hacking [60] reported that caterers, from all provision contract types, claimed that rising costs were impacting quality and menus as caterers reduced costs to compensate. This included reducing labour costs by prioritising time saving food over nutritious food, more regular use of cheaper processed food and packet mixes and reducing the variety of food offered. In addition, food costs were cut by reducing portion sizes and limiting more expensive healthy food such as protein and fish. Overcoming a lack of funding included a need to be creative by mixing high and low-cost menu items [72].

There was a large per meal price differential between the high-quality meals in Italy, which were between €5 and €6.18, and prices in the rest of Europe. Studies found prices ranged from €1.02 in Serbia to €2.28 in the UK [56]. The price charged for English school lunches was found to be insufficient to cover production costs, particularly affecting smaller schools unable to meet a breakeven rate of serving 100 meals per day [19]. Documents highlighted that the expectation and pressure for school food to generate profit in the UK and US influences policy decisions to prioritise cost savings over food quality [59,60,67]. The soil association [23] reported that successful local authority catering became unviable as profits were redistributed to education budgets.

Cost reduction was discussed as a key reason for outsourcing the school meal provision in several documents and there was an expectation that outsourcing provision would reduce costs [69–71]. However, in practice, the perceived cost effectiveness, efficiencies and economies of scale were deemed to be outweighed by higher costs for contract tendering, management, and monitoring [53,71]. One study found mixed perceptions of whether costs reduced with outsourcing, noting that ‘contractors did not always keep their promises’ [71]. Conversely, Robinson [69] found that costs and deficits were reduced after contracting out services, but the reduction was only enough to bring them in line with food authorities not using contractors. A 2013 report also stated that the provision contract type did not determine the cost of providing the meal, as schools with an in-house provision had a similar overall cost to local authorities, despite catering for much lower volumes [19]. However, there was no comparable data for outsourced private caterers [19]. RSM Ireland [63] noted that excess profits could be made by corporate providers at the expense of the public purse, however providers needed an adequate economic return to ensure quality was maintained for the lifetime of the contract.

### Nutritional quality and guidelines

Most of the papers (n = 10) [50,53,55–58,65,67,72,74], discussed **nutritional quality or guidelines**; however, there was inconsistency in the markers of nutritional quality used. Measures included compliance with standards and guidelines, which differed by country for items such as content/ servings of fruit and vegetables, fish, whole grains, high fat meat and dairy, sodium content, energy, protein, fat, vitamins and minerals, fried dishes, red meat content, processed foods, pulses and starches, pastries and cakes, confectionary, desserts and added sugars. Two papers [66,67] specifically noted the difficulty in defining and accurately measuring nutritional quality within the complexity and practical difficulties of school food provision.

Factors influencing the nutritional quality of meals in the different meal provision contract types were addressed in some studies, with in-house providers noted to be affected by a lack of resources, nutritional menu planning expertise and administrative flexibility and private contractors noted to be affected by contractual ambiguity, unsuitable selection processes and management burden [53,71]. However, the results relating to quality outcomes were also inconsistent. Three studies found no significant evidence linking the provision contract type with the markers of nutritional quality tested [50,56,74]. Three studies found that in-house facilities performed better than outsourced catering. One related to reduced sodium levels through replacing ‘heat and serve’ models with scratch cooking [65]. Two found that outsourced caterers had higher levels of operating, contract management, and overhead costs and an overall focus on reduced costs which led to including less of the expensive foods such as fruit and vegetables [53,72].

Included reports indicated that the quality of food from outsourced catering was impacted through offering cheaper, more palatable, but less healthy alternatives in competition with the more expensive healthy options to influence consumption patterns prioritising profit over health [23]. Conversely, one study found that when considering the four most important nutritional quality criteria, as defined in national guidelines and relating to fruit vegetable and meat content, outsourced canteens performed better [53] which could indicate a more commercial ability to prioritise contractual compliance. Moreover, Italian schools, which mainly outsourced their provision, were more likely to serve nutritionally calculated meals and recommend healthier choices than Finnish schools which had lower levels of outsourcing [51].

The use of commercial market forces and practices to influence choice in New York, such as making healthy food resemble fast food, creating a brand for school food and marketing the food to the children as ‘customers’ was said to raise the nutritional value of the food [64]. However, more commercialised food and a higher prevalence of Ultra Processed Food (UPF) in privately catered schools compared to state catered schools was an indicator of reduced nutritional quality elsewhere [49].

Two studies found that higher levels of **organic produce** were an indicator of better nutritional quality due to the increase in fruit and vegetable content and additional training needed for creativity in menus to accommodate the more expensive ingredients [50,53]. However, there was no robust evidence that organic procurement policies led to healthy eating or compliance with nutritional recommendations in the two studies addressing this topic [51,52].

### Disease

In terms of **chronic disease,** the papers only addressed obesity, overweight and hypertension and findings were inconsistent. Two studies found that the characteristics of the school food environment, including the provision type, were associated with overweight and obesity with lower levels present when meals were prepared in-house [49,73]. However, one study found that hypertension was higher in those schools preparing meals in house [49]. Conversely, Anderson et al., [66] found no evidence of obesity rates being impacted by provider contract type. The authors noted that this could be because all models in the study (healthy, standard, in house) were subject to the same calorie requirements. One study noted that the association between increased body weight in children attending schools that outsourced meals may be due to competition between outsourced caterers to make their meals more palatable to children compared to school nutritionists prioritising health over taste [73].

### Academic achievement

Only one study measured the impact of healthy school meal provision on academic results, finding that schools contracting with a healthy meal vendor achieved modestly better academic results than schools with in-house catering and the impact was larger for disadvantaged children [66]. Some reports and studies noted that schools prioritised academic results over quality food provision, for example, through reducing the length of lunch breaks, reducing funding or using school meal budgets to subsidise education budget shortfalls or outsourcing the provision to concentrate resource on the core education provision [19,23,67,68,71].

### Other factors influencing quality and outcomes

**Other factors** which were not dependent on provision contract type were also noted to influence the quality and outcomes from school meals. These included leadership attitude to food provision [19], inability to recruit and retain experienced staff [59,71], and administrative burden [69,70].

The impact of **culture** was highlighted in two studies [52,58] and one book [64]. Despite extensive public funding and resources devoted to school meals in Copenhagen, only 7% of pupils bought a school lunch due to Danish cultural preferences for packed lunches [52]. Similarly, a cultural prioritisation of education over school food in the UK has led to a reduced focus on quality [64]. The stronger food culture in Italy was felt to underpin the quality of the school food provision, which also incorporated environmental credentials, curriculum compatibility, local cultures and traditions, freshness & organic provision [64].

Several documents addressed the complexity of **policies and regulation** for the provision of school food of an appropriate nutritional quality, which were found to be a challenge for providers and, consequently, led to a lack of implementation and compliance. Key issues included resource constraints, lack of understanding, and a lack of adequate monitoring or consequences for non-compliance [50,53,55,60,64,67,70]. Some studies found that the existence of policies, regulations, and guidelines per se did not influence outcomes [19,52,53]. One study found that the provision contract type did not impact compliance with state menu guidelines which was low for all provision contract types tested [74]. Concerns over the ability to understand regulations and demonstrate compliance was a key reason for outsourcing provision and a robust standards regime was a priority for ensuring nutritional quality [56,70,71]. The monitoring of compliance was found to vary considerably. Italy maintains its high-quality standards with strict compliance monitoring in every school [54,64]. In contrast, England, Wales, Australia, and USA were found to have no compulsory compliance monitoring in place [59,70,74].

The difficulty of increasing **child acceptability** of healthy meals in a food environment where children prefer fast food to the compliant reduced salt, sugar, and fat healthy food was noted regardless of the provision contract type [71]. Moreover, Tregear et al., [56] found that the best nutritional quality Italian menus also generated the most plate waste (38%) and the Croatian menus with the highest level of noncompliance with nutrient standards generated the least plate waste (12%) indicating that the nutritionally balanced meals may not be appealing to the children. One report noted that healthier menus reduced the number of children taking a school meal in secondary schools but increased the number of children taking a school meal in primary school [59].

## Discussion

The aim of this scoping review was to establish what is known about the types of contracts that exist for the procurement of school meals and to identify existing evidence that links these provision contract types with the quality of food provided and the outcomes for school children. Although these findings highlight a lack of consistent evidence, they indicate that good practice can exist in each of the different school meal provision contract types of private caterers, in-house and local authority provisions. The wide-ranging differences in results and opinions suggests that the individual choice of provider may be more influential than the provision type for the quality of school meals. The decline in uptake and quality of school food and the rise in children’s health issues following the devolution of school food provision gives rise to a larger debate over whether the provision of school meals should be a commercial enterprise for profit, or a welfare service for the health and wellbeing of the nation, or if both can be possible [22]. Moreover, the magnitude and urgency of the population health crisis necessitates a solution that can drive change on a population scale.

The paucity of substantive peer reviewed academic research linking school food procurement contract type with food provision was not surprising given that a recent systematic review on the much broader terms of ‘public procurement’ and ‘food’ yielded only 63 articles [20]. Consultants RSM Ireland [63] also noted a lack of quality literature. The low number of academic studies returned and a lack of primary focus on provision contract type makes it difficult to draw robust conclusions on outcomes based on consistent evidence from several different sources.

It is commonly agreed that the quality of our diet is important to health [75] but this research highlighted a lack of a common agreement on, or definition of, **quality** with regards to school food. This makes it difficult to establish and understand the impact of different provision contract type on this key outcome for stakeholders. Most studies based their definition on guideline or standard compliance which varied per country, whilst there were some common themes such as level of fat, sugar, fruit and vegetables (though the requirements differed in each case). The methods used to define quality in the research were often subjective, such as opinions on compliance or comparison to mean values in the same study rather than to independent data [66,74].

Preparation of meals off site has been linked to ultra processed food content and poor quality [49]. However, the high-quality Italian school food system was shown to prepare food off site at central hubs [54] which would indicate that the issue relates to preparation method rather than the physical location.

School caterers have a powerful influence over the food served to, and consumed by, children and are therefore a key contributor to improving diet related outcomes for children. A set meal price for primary school meals in England precludes caterers from using pricing policies to encourage healthier choices. However, there are local policy opportunities for caterers who prioritise healthiness through using other methods to positively influence health, for example by placing the fruit and vegetables at the front of the servery in attractive configurations [76]. In addition, caterers can promote increased vegetable and fibre consumption with innovative ways of incorporating vegetables and wholegrains into meals where skilled staff and facilities allow [77].

Plate waste, found to contain mainly nutritionally important vegetables, was at higher levels in the better-quality Italian menus and lowest in the less nutritionally compliant Croatian menus [56] raising an important difference between provision and consumption and further research is needed to understand whether the difference in provision contract type influenced that outcome. These findings, which indicate that children prefer the less healthy processed fast food, highlight the difficulty for caterers to compete when offering a healthy menu in an obesogenic food environment geared towards convenience and manipulated palatability [56,71]. There is a need to consider the power and importance of including the child in the design of any solution to ensure acceptability [13].

The research highlighted the **complexity and fragmented nature** of school meal provision. Whilst positive outcomes were seen in all provision contract types Morgan and Sonnino [64] noted that where the local government service is good, its greater reach provides a more effective vehicle for reform. If outsourcing, the appointment of a catering contractor who would invest and commit to improvement has been considered key to the success of school meal provision [62]. The lack of consistency in provision type is exacerbated by a lack of local government influence or control and no central government department having responsibility for food [78]. In addition, compliance with School Food Standards is not yet measured, monitored, reported on, or enforced. All this means that a holistic systems solution is difficult to initiate, fund, action, monitor and evaluate [22,79–81] and therefore improving school food is often not prioritised [13]. Whilst the UK government has mandatory buying standards for food and catering services, they do not apply to schools and the nutrition requirements can be overridden if they are not cost effective [82].

The importance of robust and detailed **contractual terms and specifications** focused on supporting beneficial health, academic and financial outcomes was another key theme of the studies in this research. Since devolution in the 1980s, the ultimate responsibility for school food in England rests with Head Teachers and Governing Bodies [19]. School leaders are usually educational experts and may not have the time, resources, legal knowledge, or negotiation skills, to agree and manage complex procurement contracts and tendering processes [83]. Similarly, the skills of public sector officials in negotiating and managing contracts may put them at a disadvantage when dealing with large corporate providers with more extensive commercial experience and resource. Consequently, some schools are committed to onerous contracts with commercial suppliers written to fulfil commercial objectives of financial viability and shareholder value, which take priority over quality food provision for children [19].

The **economics** of school food provision were also shown to be complicated by conflicting priorities of profit and quality, and a requirement for school food to be a profit generating contributor to school budgets [19,20,59,64,67,68,71]. Much of the research pointed to issues of low budgets and inadequate spend affecting quality, but did not provide comprehensive, recent, detailed costings to evaluate the extent of the problem or potential solutions. The costings available were mainly for public sector provisions and the lack of transparency of private caterers led to consultants RSM Ireland recommending an open book system where costing information is made available for review by the state to ensure excessive profits are not being made by private sector caterers on school meal provision [63]. The one example of private sector catering being transparent on costs came from Italian stakeholders working together to agree a level of pricing and cost to support high quality food without generating excessive profits [64]. The issue of low budgets has had a significant impact on quality and unless more children take a school meal to reduce the fixed cost per meal, or prices or subsidies are increased, there is a need for school cooks to be highly skilled and creative to produce high quality food on a low budget [72]. The typical UK catering budget for school food ingredients of just 60p per pupil facilitates quality and nutrition being neglected in favour of lower cost alternatives [17,84]. Ultra processed foods (UPF), high in salt, refined carbohydrates, sugar, and fats and low in fibre are, on average, three times cheaper than healthier foods and, it is therefore unsurprising that, they make up 73% of primary school lunch calories when budgets are tight [79,85].

The quality and nutrition of school lunches in primary schools is largely driven by the caterer, with a set meal price charged of approximately £2.50 for a full meal, and children usually limited to 2 or 3 meal choices. The margins on school meals are low and without the ability to increase prices, caterers usually maintain profit margins by reducing the quality [60]. This impacts uptake and once uptakes fall below 55-60% the service fails to break even, and a vicious cycle ensues requiring further cuts and reductions to quality [22].

The impact of economies of scale of the schools on financial viability was highlighted [62]. The more profitable large schools were able to take advantage of attractive deals from private caterers wanting to win these lucrative contracts and some large schools brought the service in house to generate profit [62]. This left local authorities to subsidise small loss making schools without the budget to do so [23]. A one size fits all approach may not be suitable with additional compensation required for more expensive provisions such as small rural schools [63]. The fragmented provision and removal of the local authority role in the allocation of funding to schools makes it difficult to manage the economics of the area as a whole. Historically this was done by using profits from the larger schools to subsidise the smaller loss making schools and improve school food quality rather than for financial gain or subsidisation of other budget areas such as education [19,64].

Without fully understanding how the current complex and fragmented school meal provision landscape influences outcomes, it is difficult to develop an efficient and effective change strategy. This gap in knowledge may provide some explanation for the current situation in England, where despite the well-researched potential health benefits, school food is not a policy priority [64,78].

### Recommendations

More research is needed to inform robust regulation, monitoring and standards to improve outcomes from school food. Furthermore, research is needed to establish whether a particular provision contract type is more suited to leverage the magnitude of public sector spend on school meals to simultaneously impact environmental, social, and economic objectives. Additionally, many shortcomings in areas of good practice were identified which would benefit the design of future policy and practice relating to the provision of school meals. For example, school leaders and public sector procurement officials should have access to specialist commercial support to design, tender, negotiate and manage detailed and binding school food contracts. However, to optimise the benefit of this, there needs to be a clear, current and mandatory definition of school food quality to include in the specifications.

Additionally, work to fully understand the current cost of providing a good quality, nutritious school meal is needed which does not rely on the use of cheaper ultra processed food for financial viability. An adequate economic return for providers which is transparently monitored and reported and does not result in excessive profits at the expense of the public purse is needed. The model should adapt to differing sizes and locations of school which affect profitability and ‘ring fence’ funding for meal provision to prevent use for other budget areas. Further, the current model of voluntary participation of children in school meals in the UK should be considered. This contrasts with some other European countries which require all children to have a school meal or provide universal free school meals to better influence dietary health by enabling caterers to focus on providing health supporting meals rather than competing for business against heavily marketed, more appealing alternatives in an obesogenic food environment.

### Strengths and limitations

The robust and systematic nature of the search strategy provides comprehensive and reproducible data on what is known about contract types for the provision of school meals and their impact on outcomes for children. The inclusion of a narrative synthesis to explore the relationships will better inform policy and practice. However, it should be noted that most studies were not specifically designed to address the differences in outcomes by provision contract type and therefore may not be ideally suited to address the research question. Less than half of the documents were academic peer reviewed studies. The grey literature reports written for advocacy organisations have been written with a specific purpose in mind, which may or may not include all available information. The findings in some studies may not be generalisable, as some geographic areas may not translate to the English cultural system. In line with usual scoping review procedure [24], the quality of the included studies was not evaluated, however, it should be noted that some studies were very small [55,56,65,71], others were limited by missing and incomplete data [51,52] and others used subjective measurement methods [66].

## Conclusion

There was limited research linking school food procurement contract type with food provision for primary school children in England. However, results indicate that good practice is possible across different provision contract types which broadly fall into the categories of private caterers, in-house caterers and local authority provisions, albeit with further complexities. Although the research shows that differences in outcomes exist by procurement contract type, findings were inconsistent and do not support robust conclusions on the benefits of using any one particular contract provision type over another, indicating that the individual choice of provider may be more influential than the provision type. The grey literature and books point to a system with clear potential to impact health on a population scale through school meals. However, the complex and fragmented nature of the system and the underlying conflict between cost and quality makes it difficult to define an efficient and effective strategy on a population scale. There was no clear definition of food quality and compliance with guidelines was often used as a benchmark which makes the widescale lack of compliance found in most studies concerning. Given the magnitude of public sector spending and the need for urgent improvements to the dietary health of the nation, this presents a significant gap in our knowledge.

## Supporting information

S1 File Database search terms. (DOCX)

S2 File Preferred Reporting Items for Systematic reviews and Meta-Analyses extension for Scoping Reviews (PRISMA-ScR) Checklist. (DOCX)
