# Supplementary material for: A scoping review of the existing evidence linking school food procurement contract type with school food provision
Source: PLoS One. 2025 Mar 19;20(3):e0305685. doi: 10.1371/journal.pone.0305685 (PMC11922227; doi:10.1371/journal.pone.0305685)
Supplement: S1 File — (DOCX) [file pone.0305685.s001.docx]

**S1 File: Database search terms**

Sample search criteria and results

Database: Ovid MEDLINE(R)

Search Strategy:

--------------------------------------------------------------------------------

1     child*.mp. (2772380)

2     education*.mp. (1189586)

3     school*.mp. (386366)

4     2 or 3 (1423910)

5     junior*.mp. (23927)

6     infant*.mp. (1400302)

7     elementary*.mp. (32808)

8     primary*.mp. (1996898)

9     middle*.mp. (5009355)

10     high*.mp. (9224204)

11     second*.mp. (2327347)

12     5 or 6 or 7 or 8 or 10 or 11 (12680962)

13     meal*.mp. (86188)

14     dinner*.mp. (4650)

15     menu*.mp. (7204)

16     cater*.mp. (9186)

17     lunch*.mp. (8493)

18     13 or 14 or 15 or 16 or 17 (107760)

19     procure*.mp. (34671)

20     purchas*.mp. (44051)

21     contract*.mp. (404104)

22     buy*.mp. (14753)

23     19 or 20 or 21 or 22 (491208)

24     food*.mp. (786389)

25     nutrition*.mp. (473466)

26     diet*.mp. (893041)

27     health*.mp. (4692475)

28     BMI*.mp. (202933)

29     qualit*.mp. (1933794)

30     uptake*.mp. (457098)

31     absence*.mp. (704735)

32     attainment*.mp. (28979)

33     result*.mp. (12630300)

34     24 or 25 or 26 or 27 or 28 or 29 or 30 or 31 or 32 or 33 (16710012)

35     1 and 4 and 12 and 18 and 23 and 34 (241)
